# Impact of nodular calcification in patients with acute coronary syndrome (ACS) treated with primary percutaneous coronary intervention (PCI)

**DOI:** 10.1186/s12872-022-02551-7

**Published:** 2022-03-14

**Authors:** Abigail Demuyakor, Sining Hu, Ekaterina Koniaeva, Minghao Liu, Ziqian Weng, Chen Zhao, Xue Feng, Luping He, Yishuo Xu, Ming Zeng, Wei Meng, Yanli Sun, Boling Yi, Zhanqun Gao, Yuhan Qin, Haibo Jia, Gary S. Mintz, Bo Yu

**Affiliations:** 1 Director of Department of Cardiology, The Second Affiliated Hospital of Harbin Medical University, Director of The Key Laboratory of Myocardial Ischemia, Chinese Ministry of Education, 246 Xuefu Rd., Harbin, 150086 China; 2 Cardiovascular Research Foundation, New York, NY USA

**Keywords:** Nodular calcification, Acute coronary syndrome, Percutaneous coronary intervention, Optical coherence tomography

## Abstract

**Background:**

Calcified plaque is thought to adversely impact outcomes after percutaneous coronary intervention (PCI). This study sought to evaluate the impact of nodular calcification in patients with acute coronary syndrome treated with primary percutaneous coronary intervention.

**Methods:**

Using optical coherence tomography (OCT), 500 culprit plaques with calcification were analyzed from 495 acute coronary syndrome (ACS) patients on whom PCI was performed. Based on morphology, we classified calcification into two subtypes: nodular calcification and non-nodular calcification. Nodular calcification was defined as protruding mass with an irregular surface, high backscattering, and signal attenuation while non-nodular calcification was defined as an area with low backscattering heterogeneous region with a well-delineated border without protrusion into the lumen on OCT.

**Results:**

Calcified culprit plaques were divided into nodular calcification group (n = 238) and non-nodular calcification group (n = 262). Patients with nodular calcification were older (*p* < 0.001) and had lower left ventricular ejection fraction (*p* = 0.006) compared to patients with non-nodular calcification. Minimum stent area (5.0 (3.9, 6.3) mm^2^ vs. 5.4 (4.2, 6.7) mm^2^, *p* = 0.011) and stent expansion (70 (62.7, 81.8) % vs. 75 (65.2, 86.6) %, *p* = 0.004) were significantly smaller in the nodular calcification group than in the non-nodular calcification group. Stent under-expansion was most frequent (*p* = 0.003) in the nodular calcification group.

**Conclusion:**

This study demonstrate that the presence of nodular calcification is associated with a smaller minimum stent area and a higher incidence of stent under-expansion. Lesions with nodular calcification may be at risk of stent under-expansion.

## Introduction

Percutaneous coronary intervention (PCI) is a widely used treatment for calcified coronary lesions, which is frequently associated with increased risk of periprocedural complications and worse clinical outcomes such as target lesion revascularization (TLR) and stent thrombosis [1, 2]. Hence, the PCI approach for calcified lesions remains a challenge even in the drug-eluting stents (DES) era. Nodular calcification is defined as a protruding mass with an irregular surface, high backscattering, signal attenuation with an intact fibrous cap on optical coherence tomography (OCT) [3–5]. Recently, Kobayashi et al. reported that the amount and extent of coronary calcification as assessed by OCT were associated with stent expansion and stent eccentricity [6]. In addition, an OCT-based calcium scoring system was recently developed to predict stent under-expansion and to identify lesions that would benefit from plaque modification before stent implantation [7]. However, the response and impact of nodular and non-nodular calcification to stent implantation remains unclear. In this study, we sought to assess the impact of nodular and non-nodular calcification in patients with acute coronary syndrome (ACS) treated with primary percutaneous coronary intervention (PCI).

## Methods

### Study population

**Fig. 1:**
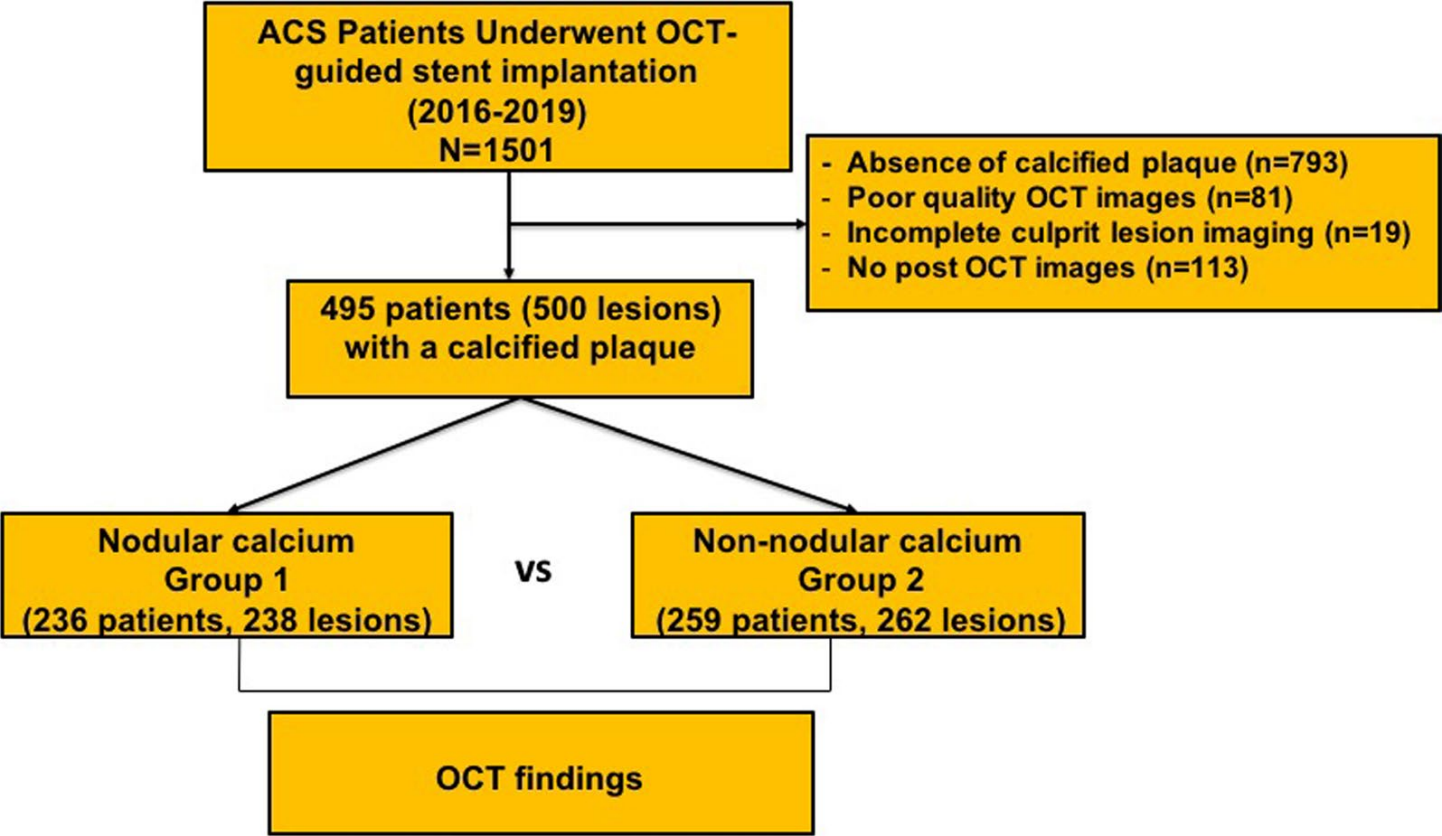
Study flowchart

The statistics show that from a total of 1501 patients with ACS who underwent OCT-guided stent implantation between January 2016 and January 2019, 708 patients had calcified plaques at the culprit lesion. Further, among the 708 patients with calcified plaques at culprit lesion, 113 were excluded because of no post-procedural OCT imaging, 19 were excluded for incomplete culprit lesion imaging, and 81exlcuded for poor image quality. Finally, 495 patients were included in the final analysis. The study flowchart is as shown in Fig. 1, and the diagnosis of ACS includes ST-segment elevation myocardial infarction (STEMI) and non-ST-segment elevation acute coronary syndrome (NSTE-ACS) [8–10]. STEMI is defined as persistent chest pain for at least 30 min, arrival at the hospital within 12 h from symptom onset, with a 12-lead electrocardiogram (ECG) changes (ST segment elevation > 0.1 mV in ≥ 2 continuous leads or new-onset left bundle branch block) and elevation of cardiac biomarker (creatine kinase-MB or troponin T/I). NSTE-ACS includes non-ST-segment elevation myocardial infarction (NSTEMI) and unstable angina pectoris (UAP). The NSTEMI is defined as ischemic symptoms in the absence of ST-segment elevation on the electrocardiogram with elevated cardiac marker levels. UAP is defined as the presence of newly developed/accelerating chest symptoms on exertion or rest angina within 2 weeks of presentation without biomarker release. A culprit lesion was identified based on abnormal manifestations of electrocardiographic, coronary angiography and cardiac ultrasound. ﻿Further, the demographic, laboratory, and clinical data, as well as angiographic and procedural data were evaluated. ﻿In addition, the pre- and post-procedural OCT findings were assessed. Moreover, all patients underwent primary PCI within 12 h of symptom onset. The study protocol was performed according to the relevant guidelines and regulations of the Declaration of Helsinki, and was approved by the Institutional Review Board (Ethics Committee) of the 2nd Affiliated Hospital of Harbin Medical University (Harbin, China). Also, all patients provided written informed consent to participate.

### Coronary angiography analysis

The angiographic images were analyzed using a quantitative coronary angiogram analysis program (CAAS 5.10.1; Pie Medical Imaging BV, Maastricht, the Netherlands). ﻿Also, lesion location, minimum lumen diameter (MLA), reference lumen diameter (RLD), diameter stenosis (DS), and the initial thrombolysis in myocardial infarction (TIMI) flow were measured by an independent investigator who was blinded to patients’ clinical information. This is done to avoid bias in the study and to ensure accuracy.

### Optical coherence tomography acquisition and analysis

OCT imaging of culprit lesions was acquired with the C7-XR/ ILUMIEN OCT system (Abbott Vascular, Santa Clara, CA, USA). Aspiration thrombectomy (Export® aspiration catheter; Medtronic, Santa Rosa, CA, USA) prior to OCT imaging was allowed in patients with large occlusive thrombus or TIMI flow grade < 1. The OCT images were digitally archived to database and analyzed in the imaging core lab by two experienced investigators (A.D and E.K) who were also blinded to patients’ information. When there is discordance between the two investigators, a consensus was obtained from experienced investigators (S.H and H.J). All measurements were performed according to previously established consensus and guidelines [11–13]. Also, the proximal and distal references were identified as the sites with the largest lumen area within a 10-mm to the plaque, and the mean reference lumen area was calculated. The minimum lumen area was identified along the length of the culprit lesion and cross-sectional bounded by luminal border including thrombus area. The lipid arc was measured for each 1 mm in the cross-sectional view, and then, the maximal lipid arc was calculated. Also, the minimal fibrous cap thickness (FCT) was measured three times in the thinnest place to obtain a mean value, and the culprit lesions diagnosis and identification were done using established criteria [18]. Thin-cap fibroatheroma (TCFA) was defined as a plaque with lipid content in at least two quadrants, with the thinnest part of the fibrous cap measuring less than 65 µm. ﻿Also, thrombus was defined as a mass floating in or protruding into the lumen with a dimension of at least 250 µm, and calcified plaque was identified as an area with low backscattering heterogeneous region with well-delineated border underlying the plaque. Based on morphology, we classified calcification into two subtypes: nodular calcification and non-nodular calcification. A nodular calcification was defined as protruding mass with an irregular surface, high backscattering, and signal attenuation covered by intact fibrous cap, while a non-nodular calcification was defined as an area with low backscattering heterogeneous region with well-delineated border without protrusion into the lumen [4, 5, 14, 15].

**Fig. 2:**
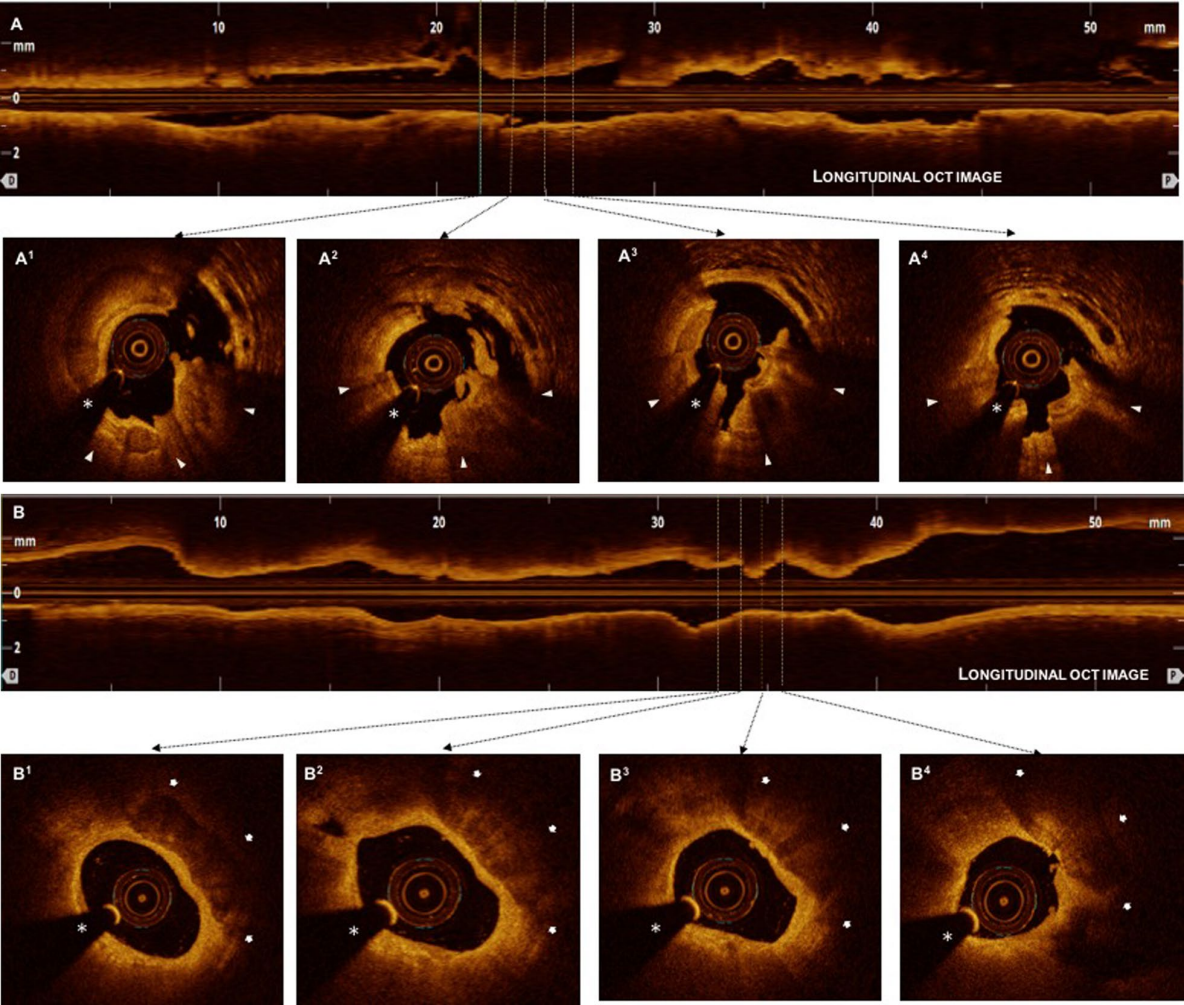
Representative optical coherence tomography (OCT) images to define nodular calcification and non-nodular calcification. **a** Longitudinal OCT image of calcified plaque. (A1-A4) Cross-sectional OCT images of nodular calcification defined as protruding mass with an irregular surface, high backscattering, and signal attenuation (arrow heads). Asterisk indicates wire artefact. **b** Longitudinal OCT image of calcified plaque. (B1-B4) Cross-sectional OCT images of non-nodular calcification defined as an area with low backscattering heterogeneous region with well-delineated border without protrusion into the lumen (arrows). Asterisk indicates wire artefact. OCT = optical coherence tomography

A representative OCT images of nodular and non-nodular calcification are presented in Fig. 2. ﻿The cross-sectional OCT images were quantitatively analyzed at 1-mm intervals, and the calcification depth was evaluated (the minimum distance from lumen to superficial calcium edge). The calcium edge is superficial if the distance between the lumen and the leading edge of calcium is less than 100 µm, and the edge is deep if the distance between the lumen and the leading edge of calcium is more than 100 µm [16]. Further, calcium score was specified as 2 points for maximum angle > 180°, 1 point for maximum thickness > 0.5 mm, and 1 point for length > 5 mm [7]. The postprocedural mean reference lumen area was defined as the mean of the largest lumen area within 5-mm of the proximal and distal stent edges, and minimum stent area (MSA), stent expansion, stent under-expansion, stent edge dissection (SED), stent strut malaposition, and tissue protrusion were evaluated using postprocedural OCT imaging data. MSA is the minimum area bounded by the stent border [12], and the percentage of stent expansion was defined as MSA divided by the postprocedural mean reference area. Stent under-expansion was defined as stent expansion < 80% [17]. Stent eccentricity index was defined as (maximal stent diameter minus MSD) divided by maximal stent diameter [6]. SED was defined as disruption of the vessel luminal surface with a visible flap at the stent edge or within 5-mm proximal or distal reference segments. SED was classified as major (≥ 60° of the circumference of the vessel at the site of dissection or ≥ 3 mm in length) or minor (any visible edge dissection < 60° of the circumference of the vessel and < 3 mm in length). Stent strut malposition clearly separated from the vessel wall by ≥ 0·2 mm is classified as a major stent (associated with unacceptable stent expansion [< 80%]) or otherwise minor. Also, a tissue protrusion was defined as tissue prolapsed between stent struts and extending inside a circular arc, connecting adjacent struts [17, 18].

### Statistical analysis

Categorical data are presented as counts and percentage, and they were compared using either a chi-square test or Fisher’s exact test, as appropriate. Continuous data are presented as mean ± standard deviations when normally distributed and as median (interquartile range) when non-normally distributed by the nonparametric one sample Kolmogorov–Smirnov test. Also, the multivariable logistic regression was used to identify independent predictor of stent expansion, and all statistical analyses were performed using SPSS, Version 18.0 (SPSS, Chicago, IL, USA). To measure significance, P-values < 0.05 were considered statistically significant.

## Results

### Baseline clinical characteristics

Finally, 500 calcified plaques in 495 patients (238 lesions in 236 patients and 262 lesions in 259 patients) were included in the current study, and the baseline patient characteristics between the two groups are summarized in Table 1. It is observed that patients with nodular calcification were older (63.8 ± 10.1 vs. 59.6 ± 10.0 years, *p* < 0.001), and more likely to have a lower left ventricular ejection fraction (57.3 ± 6.5% vs. 58.5 ± 6.2%, *p* = 0.045) compared to patient without nodular calcification. Also, the triglyceride levels were significantly increased in non-nodular calcification group (52.1 ± 28.4 vs. 60.2 ± 39.7 mg/dL, *p* = 0.015), and no significant differences in clinical presentation, history, or serum cholesterol levels were observed between the two groups.

**Table 1:** Baseline characteristics of patients

Variables	Nodular (n = 236)	Non- nodular (n = 259)	*p* value
*Patients characteristics*			
Age, years	63.8 ± 10.1	59.6 ± 10.0	< 0.001
*Gender*			
Male	146 (61.9%)	164 (63.3%)	0.738
Female	90 (38.1%)	95 (36.7%)	
Hypertension	125 (53%)	136 (52.5%)	0.919
Diabetes mellitus	59 (25%)	59 (22.9%)	0.579
Hyperlipidemia	52 (22.3%)	57 (22%)	0.934
Current smoker	100 (44.1%)	121 (48%)	0.385
Estimated GFR < 60 mL/min/1.73m^2^	10 (4.3%)	7 (2.7%)	0.340
Previous MI	16 (6.8%)	17 (6.6%)	0.903
Previous CABG	-	-	-
Previous PCI	10 (4.3%)	13 (5%)	0.703
*Clinical presentation*			
STEMI	182 (77.1%)	199 (76.8%)	0.940
NSTEACS	54 (22.9%)	60 (23.2%)	
*Laboratory findings*			
WBC count, × 10^3^/L	10.9 ± 3.2	11.8 ± 10.2	0.244
TC, mg/dL	170.9 ± 42.6	174.8 ± 38.4	0.318
TG, mg/dL	52.1 ± 28.4	60.2 ± 39.7	0.015
LDL-C, mg/dL	106.2 ± 34.4	111.1 ± 32.6	0.133
HDL-C, mg/dL	49.0 ± 11.2	47.7 ± 11.1	0.223
HbA_1C_, %	6.5 ± 1.7	6.3 ± 1.3	0.668
Hs-CRP, mg/dL	6.4 ± 5.2	6.5 ± 4.6	0.953
Peak CK-MB, U/L	215.6 ± 221.6	206.8 ± 222.2	0.684
*Echocardiographic data*			
LVEF, %	57.3 ± 6.5	58.5 ± 6.2	0.045

Values are mean ± SD or median (25–75th percentiles) or n (%)

GFR, glomerular filtration rate; MI, myocardial infarction; CABG, coronary artery bypass graft; PCI, percutaneous coronary intervention; STEMI, ST-segment elevation myocardial infarction; NSTEACS, non-ST-segment elevation acute coronary syndrome; TC, ﻿total cholesterol; TG, ﻿triglyceride; LDL-C, ﻿low-density lipoprotein cholesterol; HDL-C, ﻿high-density lipoprotein cholesterol; HbA_1C,_ hemoglobin; hs-CRP, high-sensitive C-reactive protein; CK-MB, creatine kinase-MB; LVEF, ﻿left ventricular ejection fraction

### Procedural and angiographic findings

Table 2 shows the procedural characteristics of the two groups. The two groups were not significantly different regarding the scoring balloon used before stenting, and no significant difference was detected for stent length, number of stents implanted, stent diameter, post-dilation pressure and balloon size in both groups. Further, as shown in Table 3, no significant difference was found for target vessel and initial thrombolysis in myocardial infarction in both groups. To further analyze the study groups, the post-intervention minimum lumen diameter and diameter stenosis were comparable between the two groups.

**Table 2:** Procedural characteristics

Variables	Nodular (n = 236)	Non-nodular (n = 259)	*p* value
*No. of vessel treated*			
1-vessel	230 (97.5%)	247 (95%)	0.152
2-vessel	6 (2.5%)	13 (5%)	
Scoring balloon	9 (3.8%)	6 (2.3%)	0.332
Rota ablation	-	-	-
Cutting balloon	-	-	-
Cutting balloon/NSE + RA	-	-	-
Total stent length, mm	35.7 ± 16.0	35.1 ± 15.7	0.746
Total number of stents per lesion	1.1 ± 0.3	1.1 ± 0.2	0.145
Total number of stents per patient	1.3 ± 0.5	1.3 ± 0.5	0.734
Maximum stent length per lesion	29.1 ± 6.1	28.9 ± 6.1	0.825
Maximum stent diameter, mm	3.1 ± 0.4	3.1 ± 0.4	0.265
Maximum release pressure, atm	12.5 ± 2.8	12.5 ± 2.7	0.987
Maximum post dilation pressure, atm	19.5 ± 4.2	18.9 ± 3.7	0.477
Maximum balloon size, mm	3.3 ± 0.5	3.4 ± 0.6	0.217

Values are mean ± SD or median (25–75th percentiles) or n (%)

NSE, Non-slip element balloon; RA, Rotational atherectomy,

**Table 3:** Angiographic findings

Variables	Nodular (n = 238)	Non-nodular (n = 262)	*p* value
*Preintervention angiographic findings*			
Target vessel			
Left anterior descending artery	129 (54.2%)	138 (52.7%)	0.430
Left circumflex artery	23 (9.7%)	35 (13.4%)	
Right coronary artery	86 (36.1%)	89 (34%)	
Initial TIMI			
0/1	132 (55.5%)	147 (56.1%)	0.885
2/3	106 (44.5%)	115 (43.9%)	
*Postintervention angiographic findings*			
Reference vessel diameter (mm)	2.6 ± 0.5	2.7 ± 0.5	0.052
Minimum lumen diameter (mm)	2.1 ± 0.5	2.2 ± 0.5	0.228
Diameter stenosis (%)	20.3 ± 8.3	20.4 ± 9.9	0.901

Values are mean ± SD or median (25–75th percentiles) or n (%)

TIMI, thrombolysis in myocardial infarction

### Optical coherence tomography findings

**Fig. 3:**
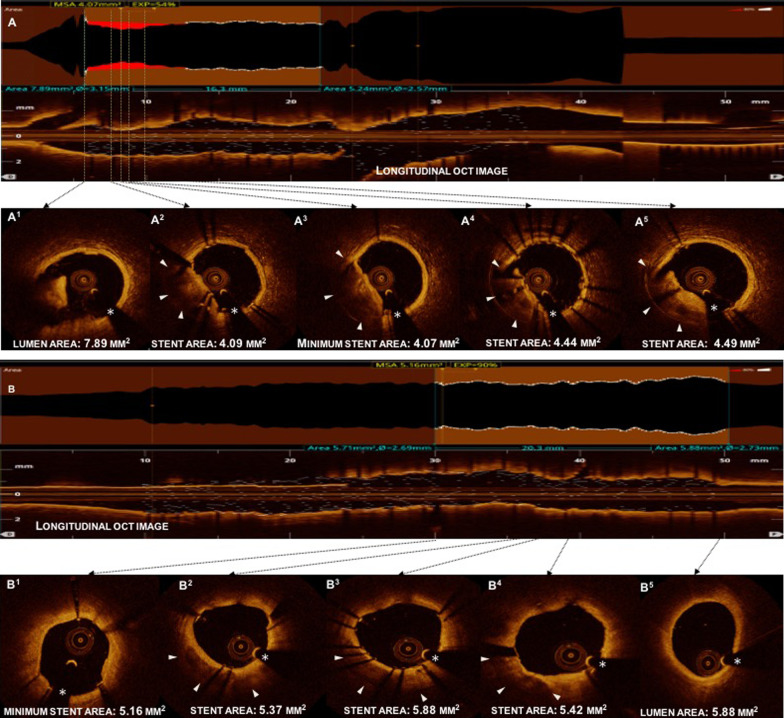
Post-stent optical coherence tomography (OCT) findings of nodular and non-nodular calcification. Case A. Nodular calcification are associated with small minimum stent area and stent underexpansion (54%). Case B. non-Nodular calcification are associated with good stent expansion (90%). Asterisk indicates wire artefact. OCT = optical coherence tomography

The preprocedural and postprocedural analysis results are shown in Table 4. The nodular calcification group had a longer lesion length (*p* < 0.001) and smaller distal reference lumen area (*p* = 0.046) compared to the non-nodular group. As compared with non-nodular calcification, calcium depth was shallow in the nodular calcification group (*p* < 0.001) and the group had a higher prevalence of superficial calcification (90.3% vs. 71.8%, *p* < 0.001). Also, the OCT-based calcium score of 4 was more frequently observed in lesions with nodular calcification (*p* < 0.001). Moreover, the minimum stent area was significantly smaller in the nodular calcification group compared with non-nodular calcification group (5.0 (3.9, 6.3) mm^2^ vs. 5.4 (4.2, 6.7) mm^2^, *p* = 0.011), the stent expansion was significantly smaller (70 (62.7, 81.8) % vs 75 (65.2, 86.6) %, *p* = 0.004) and stent under-expansion was most frequent (*p* = 0.003) in the nodular calcification group. Representative images are shown in Fig. 3, and predictor of stent expansion is analyzed in the following section.

**Table 4:** OCT findings

Variables	Nodular (n = 238)	Non- Nodular (n = 262)	*p* value
*Pre-intervention findings*			
Lesion Length, mm	31 (25.2, 38.5)	29 (22.8, 34.1)	< 0.001
Proximal reference lumen area, mm^2^	7.8 (6.0, 10.0)	7.9 (6.1, 10.3)	0.381
Distal reference lumen area, mm^2^	4.4 (3.3, 6.4)	5.2 (3.6, 6.9)	0.046
Mean reference lumen area, mm^2^	6.4 (4.9, 8.1)	6.7 (5.2, 8.5)	0.216
Minimum lumen area, mm^2^	0.9 (0.8, 1.2)	0.9 (0.7, 1.2)	0.661
Maximum lipid arc, °	288.6 (227.6, 326.9)	284.3 (211.2, 324.5)	0.418
Minimum FCT, μm	50 (40, 60)	50 (40, 60)	0.563
Thin-cap fibroatheroma	204 (86.1%)	229 (87.4%)	0.662
Thrombus	234 (98.3%)	260 (99.2%)	0.299
Calcification depth, µm	60 (40, 80)	70 (50, 122.5)	< 0.001
Superficial,	215 (90.3%)	188 (71.8%)	< 0.001
Deep,	23 (9.7%)	74 (28.2%)	
*OCT-based calcium score*			
< 4	71 (29.8%)	153 (58.4%)	< 0.001
4	167 (70.2%)	109 (41.6%)	
*Post-intervention*			
Proximal reference lumen area, mm^2^	8.3 (6.7, 10.2)	8.9 (6.8, 11.5)	0.084
Distal reference lumen area, mm^2^	5.6 (4.2, 7.7)	5.8 (4.2, 7.6)	0.592
Mean reference lumen area, mm^2^	7.0 (5.6, 8.9)	7.3 (5.8, 9.3)	0.255
Minimum stent area, mm^2^	5.0 (3.9, 6.3)	5.4 (4.2, 6.7)	0.011
Stent expansion, %	70 (62.7, 81.8)	75 (65.2, 86.6)	0.004
Stent under-expansion	172 (72.3%)	156 (59.5%)	0.003
Stent eccentricity index	0.2 (0.1, 0.2)	0.1 (0.1, 0.2)	0.228
Presence of stent edge dissection	7 (2.9%)	8 (3.1%)	0.935
Major (arc ≥ 60°, ≥ 3 mm in length)	3 (42.9%)	5 (62.5%)	0.405
Minor (arc ≤ 60°, ≤ 3 mm in length)	4 (57.1%)	3 (37.5%)	
*Presence of stent strut malaposition*			
Any	97 (41.1%)	104 (39.7%)	0.749
Major	78 (80.4%)	74 (71.2%)	0.127
Minor	19 (19.6%)	30 (28.8%)	
Tissue protrusion	232 (97.5%)	259 (98.9%)	0.207

Values are mean ± SD or median (25–75th percentiles) or n (%)

FCT, fibrous cap thickness; OCT, optical coherence tomography

### Predictor of stent expansion

Table 5 shows the results of univariable and multivariable analysis. In multivariable analysis, age, maximum post-dilation pressure, lesion length, minimum stent area, stent strut malaposition were not independent predictor of stent expansion.

**Table 5:** Univariate and multivariable predictors of stent expansion

	Univariate analysis		Multivariate analysis	
	Odds ratio (95% CI)	*p* value	Odds ratio (95% CI)	*p* value
Age, years	1.021 (1.002–1.040)	0.026	0.996 (0.958–1.036)	0.842
Gender	1.183 (0.809–1.730)	0.387		
Total stent length, mm	1.001 (0.989–1.014)	0.815		
Maximum post dilation pressure, atm	1.106 (1.004–1.218)	0.041	1.105 (0.996–1.225)	0.059
Lesion length, mm	1.026 (1.006–1.047)	0.012	0.993 (0.946–1.042)	0.769
Minimum stent area, mm^2^	0.805 (0.725–0.894)	< 0.001	0.887 (0.699–1.127)	0.327
Stent strut malaposition	1.8464 (1.261–2.754)	0.002	2.127 (0.947–4.774)	0.067
Tissue protrusion	2.425 (0.643–9.151)	0.191		
Stent eccentricity	1.783 (0.549–5.797)	0.336		

## Discussion

To the best of our knowledge, this is the first study to evaluate the impact of nodular calcification and non-nodular calcification in patients with acute coronary syndrome treated with percutaneous coronary intervention. The main findings in this study can be summarized as: (1) Minimum stent area and stent expansion were significantly smaller in the nodular calcification group; (2) Higher incidence of stent underexpansion was associated with nodular calcification group; (3) Nodular calcification frequently showed superficial calcium; 4) Patients with nodular calcification were older.

### Percutaneous coronary intervention of calcified plaques

Smaller minimum stent area and stent underexpansion are associated with in-stent restenosis and stent thrombosis following stent implantation [19–21]. Calcified plaque may adversely impact the percutaneous coronary intervention (PCI) procedure by affecting the ability to effectively dilate coronary lesion and gain an acceptable lumen area. Inadequate calcified plaque preparation before stent implantation can impede stent delivery and stent expansion [22, 23]; the consequence is often incomplete stent expansion, which increases the risk of in-stent restenosis and stent thrombosis. Lesion preparation before stent implantation is a crucial component in managing calcified coronary lesions in order to facilitate stent delivery and allow optimal stent expansion. Clinical guidelines recommend the use of rotational atherectomy before implantation for severely calcified lesions that cannot be crossed by a balloon catheter or adequately dilated [24]. A randomized controlled trial of patients with complex calcified angiographic lesions was unable to clearly show the clinical advantage of rotational atherectomy before paclitaxel-eluting stent implantation compared with balloon predilation alone; therefore, balloon dilation with provisional rotablation before stenting remains the default strategy for complex calcified lesions in the DES era [25, 26]. The potential benefits of orbital atherectomy or laser angioplasty for severely calcified lesions have been recommended by other studies [26, 27]. Hence it is important to identify and evaluate different morphology of calcified lesions that may need modification before stent implantation. Little data is available on the importance of lesion modification in lesions containing nodular calcification before stent implantation. Our study showed that nodular calcification is associated with stent under-expansion after PCI and may benefit from lesion modification.

### The evaluation of calcified plaque by optical coherence tomography

Intravascular ultrasound (IVUS) and optical coherence tomography (OCT) has been increasingly used to guide percutaneous coronary intervention procedures and improve the outcome of patient with coronary artery disease after implantation of stent [28–30]. OCT can penetrate and assess the three-dimensional extent of calcium, whereas the evaluation of calcium by IVUS is limited because ultrasound is almost entirely reflected from the calcium surface. Additionally, OCT can provide precise evaluation for superficial calcification that might be related to poor stent expansion [31, 32]. Recently, Fujino et al. reported an OCT-based calcium scoring system and the risk of stent underexpansion was increased in lesions with calcium score of 4. Lesions with calcium score of 0 to 3 had excellent stent expansion, whereas the lesions with a score of 4 had poor stent expansion and aggressive lesion modification should be considered when treating them [7]. Our study reveals higher incidence of calcium score of 4 in nodular calcification group; suggesting plaque modification before stent implantation might be helpful. The amount and extent of target lesion calcification has been suggested to be an important contributing factor in stent expansion but the morphology of the calcified lesions was not reported [6]; utilizing OCT, we highlighted the impact of nodular calcium protruding into the lumen causing an inadequate stent expansion which may result in abnormal sheer stress that might be associated with smaller stent area.

### Study limitations

First, this was a retrospective, observational study with a modest number of patients. Second, our classification of calcification into nodular and non-nodular based on morphology is novel and therefore has not yet been validated. Third, in patients with TIMI flow grade 0/1, manual thrombectomy was performed to re-establish effective vessel patency, allowing safe and high-quality OCT imaging data collection at the culprit site. However, the potential effect of the thrombus aspiration catheter on superficial plaque integrity and atherothrombotic components assessed by OCT must be given serious consideration. Fourth, the analyzed cross-sections using OCT could be inconsistent between pre-and post-PCI. Finally, this study was conducted with only postprocedural, hence, a large scale with long-term follow up is required.

## Conclusion

Calcified plaque adversely impacts stent implantation and remain a challenge for an interventional cardiologist. However, the characteristics of calcification morphology may influence the extent of this impact. When considering morphology features, this study demonstrated that the presence of nodular calcification (protruding mass with an irregular surface covered by an intact fibrous cap) is associated with a smaller minimum stent area, and a higher incidence of stent underexpansion in patients with acute coronary syndrome treated with primary PCI. Lesions with nodular calcification may benefit from plaque modification (specialized balloons and atherectomy devices) before stent implantation.

## Data Availability

All relevant data and materials are included in the manuscript. The datasets will be available from the corresponding author on reasonable requests after study completion.

## Abbreviations

ACS: Acute coronary syndrome
PCI: Percutaneous coronary intervention
OCT: Optical coherence tomography
IVUS: Intravascular ultrasound
TLR: Target lesion revascularization
DES: Drug-eluting stents
STEMI: ST-segment elevation myocardial infarction
NSTE-ACS: Non-ST-segment elevation acute coronary syndrome
ECG: Electrocardiogram
UAP: Unstable angina pectoris
MLD: Minimal lumen diameter
RLD: Reference lumen diameter
DS: Diameter stenosis
TIMI: Initial thrombolysis in myocardial infarction
FCT: Fibrous cap thickness
TCFA: Thin-cap fibroatheroma
MSA: Minimum stent area
SED: Stent edge dissection
MACE: Major adverse cardiac events
